# Getting in touch: Communication in physical therapy practice and the multiple functions of language

**DOI:** 10.3389/fresc.2022.882099

**Published:** 2022-08-04

**Authors:** Birgitte Ahlsen, Anne Birgitta Nilsen

**Affiliations:** ^1^Department of Rehabilitation Science and Health Technology, Oslo Metropolitan University, Oslo, Norway; ^2^Department of Interdisciplinary Health Sciences, University of Oslo, Oslo, Norway; ^3^Department of International Studies and Interpreting, Oslo Metropolitan University, Oslo, Norway

**Keywords:** communication, physical therapy, verbal, non-verbal, patient—centered care, case study, language

## Abstract

In physical therapy, communication that actively involves the patient is seen as the foundation of patient-centered treatment. Research on communication in physical therapy highlights how patients' opportunity to actively participate is often limited by the therapists' focus on biomedical facts and clinical tasks. Few studies have explored aspects of communication in clinical practice that may promote patients' active participation. The aim of this study is to shed light on verbal and nonverbal communication used by physical therapists to get in touch with patients and how this physical and linguistic touching may contribute to encouraging patients' participation. The selected case is from a qualitative observational case study of the first encounter between a female physical therapist and a male patient with chronic neck pain. Drawing on theories about communication and the metafunctions of language, the findings highlight how the therapist's use of unfinished sentences, repetitions of the patient's own words, touch, gaze and accepting interruptions from the patient promotes the patient's participation. Demonstrations of the use of linguistic communication theory in this study may contribute to enhancing physical therapists' self-awareness around communication and how to get in touch with patients, which is a fundamental element in patient-centered treatment.

## Introduction

Patient-centered approaches have been introduced in health care practices as a way of reducing the gap between the biomedical model of disease and the patients' experiences of illness (1, 2).

In physical therapy, the growing interest in patient-centered approaches has helped highlight the relationship between therapist and patient and communication in physical therapy practice (3). Good communication is pointed to by patients as important for feeling involved in the treatment process and for their participation (4, 5). However, studies on physical therapy practice support the claim that the voice of the practitioner tends to suppress the voice of the patient's lifeworld (6–13). Josephson et al. (13) assert that while the patients seemed to engage in the consultation from both a clinical and interpersonal perspective, the physical therapists were more concerned about what mattered to them clinically. Similarly, work by Hiller et al. (6, 7) underscores how communication between physical therapist and patient was predominantly characterized by a “practitioner-centered” approach, because of the way communication was structured and because the therapists mainly focused on the physical aspects of the patient's health problem. However, Hiller et al. nuance their findings by emphasizing how touch, as a form of non-verbal communication resource in physical therapy practice, seemed to promote a patient-centered approach (ibid). In general, the literature on patient centeredness in physical therapy commonly emphasizes the fact that physical therapy practice is heavily rooted in the culture of medicine, and that physical therapists often feel insecure when moving beyond their field of expertise—i.e., the physical body—and communicating with patients about their social and emotional life (8, 11, 14, 15). Thus, previous research in physical therapy concludes with a call for empirical research that may help theorize clinical conversations and help enhance physical therapists' self-awareness around communication, which is a fundamental element in the treatment of patients (7, 14).

With its focus on getting in touch this study aims to contribute to the discussion on communication in physical therapy and begin bridging the knowledge gap by focusing on aspects of communication in clinical practice that can a) promote patient participation and b) facilitate opportunities for patients to talk about what is important to them. Our focus is on patients with chronic muscle pain. The complexity of chronic muscle pain has particularly challenged standard biomechanical physical therapy approaches and shifts the focus to patient-centered approaches and communication between therapist and patient (14, 16).

Based on a first encounter between a patient with chronic muscle pain and a physical therapist in primary health care, the question explored in this study is: How do physical therapists use verbal and nonverbal resources to get in touch with the patients, and how do these resources help encourage patients' participation in the physical therapy encounter? In this article, touch refers to both the physical touch and the relational touch, the contact that occurs in communication.

## Communication theory: Metafunctions of language

Three metafunctions are important components of human communication; following Halliday (17), these are the *ideational*, the *interpersonal*, and the *textual*. The *ideational metafunction* concerns the content—our language use as representations of the world in the broadest sense and includes our own consciousness [(17): 66].

The *interpersonal metafunction* concerns touch and getting in touch. This metafunction is about how language use touches the listener and forms contact and a relationship between speaker and listener. Through the use of verbal and nonverbal resources, we get in touch with each other and establish different types of relationships. The interpersonal metafunction is thus all that may be understood by the expression of our own personalities and personal feelings on the one hand, and forms of interaction and social interplay with other participants in the communication situation on the other hand [(17): 66].

The third metafunction, the *textual*, creates coherence between the ideational and the interpersonal metafunctions and concerns the flow of information in a text: i.e., how humans use language to create coherence, for example in a conversation or a letter. The textual metafunction is the component that enables the speaker to organize what he is saying in such a way that it makes sense in the context and fulfills its function as a message [(17): 66].

The three metafunctions presented above are always present when we communicate. In our analysis, we will use the concepts of the metafunctions to describe what is going on in an encounter between a physical therapist and a patient. More specifically, we will describe how the physical therapist makes use of her knowledge and experience in her interaction with the patient with a reference to the ideational metafunction. Furthermore, we will describe the textual function and draw the readers' attention to how sense is made between the physical therapist and the patient. In our analysis, we also describe how the expression of personal information and personal feelings are used in getting in touch and establishing a relationship in the encounter, with a special focus on the physical therapist's use of verbal and non-verbal resources. Worth noting here is that the division of metafunctions is made for the purpose of analysis. In some examples, some metafunctions may be more distinct than others, but in practice, all three are always simultaneously present in interactions between people.

## Materials and methods

### Case study

A case study is a study of real-world practice and a research methodology that enables the production of deep, rich, and complex insight into the practice under study. It is a method with which one can learn about a complex instance through description and contextual analysis. In our case study, we have focused on communication: more specifically, on the physical therapist's use of verbal and nonverbal resources to get in touch and encourage the patient's participation. We also wanted to introduce linguistic theory about communication, and we believed that the linguistic theory of language metafunctions would be useful, not only for our study, but also for the field of physiotherapy in general. Because we started out with a theory, we adopted a qualitative descriptive single case study, described by Yin (18), to explore communication and interaction between physical therapist and patient. According to Yin, descriptive cases require that the investigator begin with a theory to keep the study within manageable proportions [(18): 39]. An advantage in this case study was the inside perspective of the physical therapist (the first author) and the outsider perspective of the linguist (the second author).

The results of our case study are both descriptive and theoretical in the sense that questions were raised about why the instance occurred as it did, and what may be important to explore in similar situations. In other words, we used a theoretical framework to establish a logic that might be applicable to other situations [(18): 18]. The case study approach proved suitable for answering questions about how best to communicate to get in touch with and encourage patient's participation, enabling us to learn what works (and what does not). Our overall aim with this approach was to enrich our understanding of communication in physical therapy.

Every consultation is different, and the ways in which different physical therapists communicate with their different patients are contextual. Nevertheless, we believe the analysis of this case study may be useful in raising physical therapists' awareness of their communicative practices and thus be applicable beyond the particular context of this case study. Furthermore, the case we studied represents the voice of individual actors, but also the voices of relevant groups—i.e., patients and physical therapists—and the interaction between them.

### Ethics

The study was conducted in line with the Declaration of Helsinki and was approved by the Norwegian Social Science Data Services (ref. 38954). Participating physical therapists and patients provided written informed consent. Throughout the research process, the researchers sought to ensure the integrity of the patients and physical therapists: for example, by anonymizing and concealing the identity of the participants and by presenting information in a sensitive manner.

### Recruitment and participants

The case was selected from a total of nine observational studies of the first encounter between physical therapists and patients with chronic muscle pain. All the physical therapists who participated in the project were based in primary health care and were running their own private business with financial support from the government. The study aimed for strategic sampling to create heterogeneity in terms of sex and professional expertise (19). Information letters were sent to a number of physical therapy clinics located in primary health care settings; while some were chosen at random, others were selected on the basis of their reputation or because they formed part of the first authors' wider network.

All the participating physical therapists were of Norwegian ethnicity. Four were men and five were women; all were in their forties or fifties. Located in seven different institutes spread across five different regions, all were highly qualified, experienced, and enthusiastic practitioners. Eight of the nine were specialists in either manual therapy or psychomotor physical therapy.

Patient participants were recruited by the therapists, who passed on information verbally and via an information sheet. Of the nine patients recruited as participants, four were women and five were men. The women were aged between 35 and 70 years, while the men's ages ranged from 32 to 70 years. All were suffering from prolonged muscle pain.

### The case

Interactions between therapist and patient with regard to the therapist's use of verbal and nonverbal resources and the patient's response were observed in our corpus of data. From among the encounters covering both the interview and physical examination, we selected the one that appeared to have the most interesting interactional development between patient and therapist—in the sense that the patient's interaction seemed to increase during the encounter. While he initially offered short answers to the physical therapist's questions, we noticed that the patient's participation began increasing: he began talking more, used more nonverbal communication (such as smiling and laughing), seemed more engaged, elaborated more, concluded some topics and initiated others. The increase in patient's participation may be described as getting in touch, increasing contact which we will demonstrate in our analyses.

The patient was a man in his forties seeking psychomotor physical therapy because of chronic neck pain. The physical therapist, a specialist in psychomotor physical therapy, was a woman in her fifties. As the interview began, both therapist and patient were seated in armchairs, with a small table between them. The therapist regularly jotted down notes on a sheet of paper in her lap during the interview. The clinical interview lasted for about 20 min, and was followed by a 40-min physical examination.

### Analysis

We began our analysis by watching some of the videos from the material described above. We watched the videos several times, with a particular focus on the interaction and relationship between therapist and patient. We then selected one video where the relationship between therapist and patient seemed to gradually develop during the consultation. As described above, the patient shifted from giving brief answers to the therapist's questions to becoming more engaged: this included talking more, initiating new topics, leaning forward in his chair, and occasionally smiling at the therapist. This video contrasted with another video in which the therapist, who was seated in front of a computer, asked the patient a number of questions and was busy writing what he heard, but did not seem equally interested in the interpersonal relationship. Although the therapist likely got to know the patient better (from the patient's responses), the contact between them did not seem to change: the focus was on the ideational and textual metafunctions of language and to a lesser degree on the relational.

We analyzed the selected video by sitting next to each other and watching it on a large screen. We watched the video three times in total—and specific sequences several times—and each took notes. We paused the video at sequences that caught our attention, and discussed what we were watching. Our focus was on the physical therapist and what we interpreted as her means of encouraging the patient's participation. We were particularly interested in the relational aspects of her communicative style, how she gets in contact with her patient: relational aspects that could potentially contribute to the patient's participation. We also paid careful attention to the patient's response to the therapist as indications of her communication's effect on him. Focusing on the relational aspect of language is how humans seek to get in touch with each other. In our analyses, we will demonstrate how a therapist gets in touch with her patient through verbal and non-verbal touching.

## Results

Our findings show that this physical therapist uses a rich repertoire of verbal and non-verbal resources to get in touch with her patient.

As mentioned above, we also notice that the patient becomes more relaxed as the interaction progresses and participates more actively in the conversation. We interpret these observations as getting in touch. The physiotherapist communicative style touches the patient in such a way that he is encouraged to participate in the interaction. Inspired by the Language of Touch by Mirt Komel (20), we claim that communication is touching. We not only touch each other when body parts meet, but also with verbal and non-verbal resources.

Below, we demonstrate how the physical therapist gets in touch with her patient by expressing her attentiveness and encouraging the patient's participation through verbal and nonverbal resources. We begin with an example of how the physical therapist handles the patient's interruptions and continue with the physical therapist's use of repetitions, unfinished sentences, touch, and gaze.

### Interruptions

Goldberg (21) defines three types of conversational interruptions: relationally neutral interruptions, power interruptions, and rapport interruptions. Relationally neutral interruptions are intended to repair, repeat, or clarify something the speaker has just said; a power interruption occurs when the interrupter breaks in and cuts off the speaker to display social power; and a rapport interruption is done to show mutuality and empathy, and is interpreted as collaborative and cooperative. In our case study, rapport interruptions can be seen in the physical therapist's withdrawal whenever the patient interrupts her, to give space for his participation. One way that she withdraws is by accepting the patient's interruptions by terminating her own actions, which may be seen as an indirect encouragement of participation. This is thus a response in which she relies on non-verbal resources. We interpret this as a way of supporting the patient's participation in their conversation. As an example, during the clinical interview, we see that the physical therapist takes a breath, thus indicating that she is going to start talking, when the patient interrupts her and begins to talk. The therapist lets him continue talking, not insisting on her turn to talk. Her withdrawal likely encourages the patient to talk, because she is signaling that what he has to say is more important.

In another example, the physical therapist picks up a stool that she is about to move from one side of the room to the other. When the patient begins talking, she immediately terminates her action, sits down on the plinth with the stool on her lap, turns her face toward the patient, and listens attentively to what he says. When the topic initiated by the patient is concluded, she resumes and completes her action: she stands up from the plinth, walks to another part of the room, and puts the stool down. The physical therapist takes a similar approach when she is taking notes, demonstrating her attentiveness by terminating her writing, raising her head from the paper, and looking at the patient when he begins to talk.

Later, when the therapist is about to finish the examination of the patient, she moves away from the patient, who is sitting on the plinth, and fetches his shirt from another part of the room. On her way back to the patient, he begins talking. Again, the therapist immediately stops, turns her face toward the patient, and listens attentively while holding on to the shirt. When he is finished speaking and that part of their conversation is concluded, she hands the shirt over to the patient. By doing so, the therapist is demonstrating that the patient may take his time to talk.

Fetching the patient's shirt is in itself a friendly gesture and may also be interpreted as a sign that the examination is over. It is also worth noting that the therapist is contributing to equalizing the power relationship embedded in the clinical encounter when she indicates that what the patient has to say is more important than her actions. In clinical encounters, there is a difference in power between therapist and patient: the patient is the one in need of help from the therapist, and the therapist determines the framework and conditions for the clinical encounter and decides whether the patient is entitled to treatment. In the literature, the sharing of power and responsibility in clinical encounters is commonly highlighted as a central dimension of patient-centered care (2). Here, we have demonstrated how the sharing of power is expressed through the therapist's acceptance of being interrupted by the patient, terminating her own actions, and looking at the patient when he speaks.

### Repetitions

Repetition is a linguistic resource by which speakers may create content, a relationship, and coherence, thus fulfilling the three metafunctions. In other words, repetition not only ties parts of the conversation to other parts, but it bonds participants to the conversation and to each other; it links individual speakers in a conversation and in relationships, as repetition gives the people involved in the conversation an impression of a shared universe of discourse [(22): 61]—thus creating interpersonal involvement. According to Tannen (22), repeating the words, phrases, and sentences of other speakers (a) produces a conversation, (b) shows one's response to another's utterance, (c) demonstrates acceptance of others (including their utterances and their participation), and (d) gives evidence of one's own participation. Repetition also provides a resource to keep talk going, where talk itself is a show of involvement, of a willingness to interact. All of this, Tannen asserts, sends a metamessage of involvement and attentiveness. This metamessage may thus function as a way of getting in touch.

In what follows, we provide some examples of how the physical therapist uses repetition in her interaction with the patient. In our first example, we see that the physical therapist repeats something the patient has said earlier during the consultation. She is echoing one of the patient's utterances:

Patient: I used to sleep a lot on my stomach.

Physical therapist: You slept a lot on your stomach, yeah.

In echoing the patient, the patient may interpret her as attentive and interested and as accepting his utterance. In another repetition, the physical therapist is sitting beside the patient on the plinth:

Physical therapist: Let's see, if you let out your stomach toward my hand, can you do that?

Patient: We try to keep the stomach in, you know [smiling].

Physical therapist: We do that, you know [laughing], when we pass 40.

They both laugh about how they try to keep their stomachs in, and the patient likely interprets the physical therapist as appreciative of his humor. Thus, they have established a shared humorous view that likely strengthens their relationship and mutual trust. This is what we observe in our case study: that the physical therapist and the patient gradually reveal aspects of their selves to each other (such as their sense of humor), and we believe that this development in the conversation may contribute positively to the patient's participation.

Below, we present an example of repetition related to the textual metafunction, where the repetition functions as a question for clarification:

Patient: After I started working as a clerk, you might say, I worked as an engineer.

Physical therapist: Worked as?

Patient: As an engineer.

### Unfinished sentences

A conversation consists of sentences, and each sentence can be collaboratively constructed by both the speaker and the listener: this occurs when the speaker voices part of a sentence (i.e., an unfinished sentence) and the listener completes the sentence—and thus the full thought (23, 24). Below, we demonstrate the use of unfinished sentences and how these sentences function as an invitation to complete the sentence, and thus encourage the patient's participation. In this way, the unfinished sentence is related to all three metafunctions of language. In our first example of an unfinished sentence, the topic is the patient's back pain, which he had a long time ago and which he describes as the beginning of his chronic pain problem.

P: In'95, my back was so bad I went for occupational retraining. At that time, the back was my main problem. The retraining went fine, but after I started working as a clerk, I gradually began getting problems with my shoulder and neck.

T: Shoulder and neck, so the thing with the back, that's kind of…?

P: It's there too, but it's not a problem in the work context.

In the patient's response, we see that he accepts the physical therapist's invitation to complete the sentence, to enter a collaborative conversational endeavor. However, the incomplete sentence may not only function as an invitation to collaborate—with the incomplete sentence, the physical therapist is also demonstrating that she has an interest in the patient's explanation of his condition. The therapist is not guessing at what the patient is about to say, by giving him alternatives: for example, by saying, “So, the thing with the back is healed and not a problem today?” Rather, she leaves the room open for the patient to elaborate freely on his back problem.

We see another example of an unfinished sentence in the physical examination. The patient is lying on his back on the plinth and the therapist is palpating the muscles in his left arm, when the patient again mentions his back problems:

P: I had an additional period with pain in my back last year during the spring. At that time, it felt as if my feet failed, and my back was painful. So, walking downhill on uneven ground was unpleasant.

T: Okay, you lost a bit like…

P: I felt as if I lost 30% of the power.

By putting forward these open-ended unfinished sentences, the therapist is actively encouraging the patient's participation.

### Touch

In what follows, we demonstrate that touch in physical therapy can also be related to all three metafunctions of language. Here, we consider nonverbal resources (such as touch) as part of the communicative repertoire. Touch may contribute to meaning making, establishing relationships, and creating coherence in conversations. In our case study, we find that the physical therapist's touches seem to have two different main functions: one is physiotherapeutic and the other is relational. One example can be seen in a sequence where the patient is sitting on a stool and bows his head forward. The patient tells the physical therapist that there is a stiffness in his neck that continues down along his left shoulder. The physical therapist moves her hand gently from the patient's neck down to his left shoulder. This tactile move may be interpreted as a way of confirming that she has heard the patient referring to his neck and left shoulder. In this case, her touch is about meaning making and coherence. One may also interpret her touch as a comforting action, in which case it would be relational. Her touch may therefore be viewed as contributing to positive contact between the physical therapist and the patient, as she is demonstrating that she is listening and that she cares about the patient's shoulder. We also find similar examples, such as when the physical therapist moves her hand gently over the part of the body to which the patient is referring.

Another example of the multifunction of touch can be seen when the patient is standing on the floor, and the therapist alternates between standing in front of, next to, and behind him. The therapist tells the patient what she is looking for and at the same time she touches him. For example, when she says that she is looking at his posture in a standing position, she touches the patient's shoulder, hip, and knee. When the therapist says that she is looking at the position of his arms, she touches his arms, and so forth. Through her use of touch, the therapist underlines the content of the conversation (the patient's body posture), creating coherence; at the same time, she is also creating a relationship with the patient in which they are both engaged as active participants. We see that the patient actively tries to notice the areas of the body that the therapist touches (he makes small movements with his arms and head) and makes comments. For example, he says that his arms are painful and that his head feels heavy sometimes and that he needs to bend it backward to rest. He also tells the therapist about a visit to the doctor and what happened during that visit. In this sequence, then, the function of touch is to simultaneously create meaning, coherence, and contact at the same time.

In other sequences, we see that the physical therapist's touch is essentially therapeutic, used for the purpose of guiding and assisting the patient in his movements. For example, when the patient is sitting on the stool and is asked to let go of his head and bend forward, the therapist gently touches his forehead to guide the movement and support his head. The therapist then moves her hand along his back and asks him to bend forward a little more. These movements are difficult for the patient and are repeated a couple of times. Each time, the therapist adds some more touches to assist the patient and guide his movements—on his forehead, neck, back, and stomach. The therapist asks the patient what he notices. At the end of this session, the patient turns his head, looks at the therapist and says with a smile: “Didn't notice much.” Here, we interpret the therapist's use of touch as ideational and textual, and as contributing to meaning making and coherence. However, we also see the therapist's gentle use of touch as supportive care of the patient. He in turn shows that he feels safe and taken care of by making eye contact with the therapist and smilingly admitting that he did not quite follow her (i.e., “notice much”).

### Gaze

Gaze refers to the kind of interaction between participants in communication and may be used to offer or demand eye contact [(25): 117–118]. We notice that this physical therapist uses gaze very actively in her interaction with the patient. Her use of gaze seems to have several functions. First, she uses gaze to demonstrate her attentiveness and interest in what the patient says, but she also seems to use gaze actively in the interaction to ensure, maintain, and establish contact with the patient. In one example, the physical therapist stands behind the patient while examining his back. During the examination, they talk together and after a while she moves to the front of the patient, so that they are looking at each other when they talk. She stands in front of him for a time while he is sitting on the plinth, and then sits down beside him on the plinth, and they continue talking. After a while, she returns to standing behind the patient, examining his back and posture. The therapist's movements and establishing of eye contact with the patient during the examination may be interpreted as a sign that she cares about him and not just his body. Another example of the therapist's use of eye contact occurs when the patient is lying on his back on the plinth and the therapist touches one of his feet and makes some movements. She examines the patient's foot and leg and maintains regular eye contact with him by looking often at his face, as if to check that he is okay. We can also observe that when the therapist looks at the patient, he usually starts talking—making comments on what he feels and notices, and sometimes making associations with his life. As such, the therapist's use of gaze is simultaneously therapeutic (used for the purpose of examination) and relational.

## Discussion

The aim of the study was to gain knowledge about a) physical therapists' use of verbal and nonverbal resources to get in touch with patients and b) how these resources help encourage patients' participation in the dialogue/encounter. Drawing on theories about the metafunctions of language, the findings—here represented by a single case —highlight how the therapist's use of verbal resources, such as the use of unfinished sentences and repetition of the patient's words helped to get in touch with the patient and facilitate the patient's participation. In addition, findings show how the therapist's use of nonverbal resources (e.g., accepting interruptions, not insisting on her turn to talk, terminating her actions when the patient took the initiative to talk, making eye contact with the patient while talking, and actively and subtly using touch) seemed to be successful means to get in touch with the patient and encouraged the patient's active participation in the interaction.

Importantly, our findings highlight the many functions of touch in physical therapy and how the therapists' use of touch, in the sense of getting in touch, can be interpreted as related to the ideational, relational, and textual metafunctions of language.

Nicholls and Holmes (26) critically discuss how touch in physical therapy has been disciplined into a technical device, used by the therapist for a clear biomechanical rationale and consequently, how the therapists' opportunities to discuss and study the value of touch in clinical practice are limited. As a follow-up, Bjorbækmo and Mengshoel (27) describe how touch in physical therapy is seldom discussed and referred to as such, but instead exists tacitly in other concepts, such as palpation, nonverbal skills, body work, and massage. Through close observation of one case and the interaction between a physical therapist and a patient in their first encounter—focusing on the physical therapist's use of verbal and nonverbal resources and the patient's response—our findings show how touch as communication may help empower patients and facilitate their active participation in the therapist–patient interaction. As such, the therapist's use of touch may constitute an important resource in patient-centered physical therapy.

Previous research on communication in physical therapy practice has emphasized the dominance of the biomedical discourse in the clinical encounter, with therapists being narrowly focused on physical data and their own clinical tasks (6, 8, 10, 13). Our study takes a slightly different perspective, by focusing on the therapist's use of verbal and nonverbal resources in clinical practice which facilitates the patient's participation in the interaction. As such, our study adds to the existing knowledge by showing what is already being done and what therapists can attend to in their efforts to empower patients and promote their participation (e.g., unfinished sentences, repetition, accepting interruption, touch, and gaze).

The caring aspect of the physical therapist's use of both verbal and nonverbal resources in interactions with patients is clearly present in our findings. Care was demonstrated through the therapist's use of touch, gaze, and her acceptance of the patient's interruptions, which showed recognition and respect for what the patient had to say. In the interview we conducted with the patient following the consultation, he described the therapist's form of communication as caring. While patient-centeredness involves care (28), care is seldom discussed in physical therapy—and when it is, it is usually associated with communication, seen as something that is added to the treatment (29). The Dutch anthropologist Annemarie Mol argues that care is not something that is added to clinical practice (such as a friendly gesture), but is something embedded in clinical practice, through interaction and recognition (30). Good care, Mol argues, entails time spent listening to the patient, attuning to the patient's body and needs, and acknowledging the patient as a person (30). Our findings show how the therapist in this case recognized the patient through repeating the patient's words (which demonstrated that she was listening and acknowledging what he was saying) and accepting interruptions by the patient (which showed respect for what he had to say). In addition, our findings show how the physical therapist's recognition of the patient was demonstrated through pauses (which signaled that she had plenty of time for him), eye contact, and touch. Thus, following Mol, our findings show how care may be embedded in physical therapy practice, both verbally and non-verbally, and linked to getting in touch with the patient.

### Strengths and limitations

We are aware that generalizations cannot be made from a single case, in the sense that the verbal and non-verbal language resources used by the therapist may not have the same effect in the interaction between a therapist and patient in another clinical setting. However, by drawing on communication theory and conducting close observation of one case, our study may inspire some physical therapists and aid them in becoming more aware of their communication with their patients. A strength of the study is its interdisciplinary approach, and the cooperation between a physical therapist and a linguist in the analysis. Through the linguist's outsider perspective, the study highlights aspects of communication and interaction in physical therapy that may be taken for granted and go unnoticed by a physical therapist. At the same time, the physical therapist's knowledge of the field of study helped give meaning and significance to the linguist's observations. As such, we believe our study offers a valuable contribution to the discussion around communication in physical therapy, patient-centered care, and further research in this area.

## Concluding remarks

The relationship between the patient and the physical therapist is at the heart of healing—a relationship that is established through conversations consisting of verbal and non-verbal cues. In this article, we have presented an analysis of a clinical interaction between a patient and a physical therapist in primary health care. Through our analysis, we have explored how physical therapists may use verbal and non-verbal resources to get in touch with patients and how these resources may encourage patients' participation in the physical therapy encounter. Our aim is that our demonstration of the use of linguistic communication theory may enhance physical therapists' self-awareness around their communication, which is a fundamental element of patient-centered care.

## Data availability statement

The data is archived in Secured Data Repository for Research at the University of Oslo, and to protect the privacy of the participants, data cannot be shared publicly. Requests and further questions should be directed to the corresponding author.

## Ethics statement

The studies involving human participants were reviewed and approved by REK Regional Committees for medical and health research ethics (ref. 2014/739). The patients/participants provided their written informed consent to participate in this study. Written informed consent was obtained from the individual(s) for the publication of any potentially identifiable images or data included in this article.

## Author contributions

BA planned the study, conducted the observations and interviews, and submitted the paper and is responsible for the overall content of the article. BA and ABN analyzed the data material and drafted the paper together and contributed equally in developing the paper as a whole.

## Funding

Norwegian Fund for Post-Graduate Training in Physiotherapy, Grant No. 45027.

## Conflict of interest

The authors declare that the research was conducted in the absence of any commercial or financial relationships that could be construed as a potential conflict of interest.

## Publisher's note

All claims expressed in this article are solely those of the authors and do not necessarily represent those of their affiliated organizations, or those of the publisher, the editors and the reviewers. Any product that may be evaluated in this article, or claim that may be made by its manufacturer, is not guaranteed or endorsed by the publisher.
